# Genomic Analysis To Identify Signatures of Artificial Selection and Loci Associated with Important Economic Traits in Duroc Pigs

**DOI:** 10.1534/g3.118.200665

**Published:** 2018-09-20

**Authors:** Yunlong Ma, Saixian Zhang, Kaili Zhang, Chengchi Fang, Shengsong Xie, Xiaoyong Du, Xinyun Li, Debin Ni, Shuhong Zhao

**Affiliations:** Key Laboratory of Agricultural Animal Genetics, Breeding, and Reproduction of the Ministry of Education & Key Laboratory of Swine Genetics and Breeding of the Ministry of Agriculture, Huazhong Agricultural University, Wuhan 430070, P. R. China

**Keywords:** Duroc pig, Artificial selection signatures, Genome-wide association study, Economic traits

## Abstract

Identifying genetic basis of domestication and improvement in livestock contributes to our understanding of the role of artificial selection in shaping the genome. Here we used whole-genome sequencing and the genotyping by sequencing approach to detect artificial selection signatures and identify the associated SNPs of two economic traits in Duroc pigs. A total of 38 candidate selection regions were detected by combining the fixation index and the Composite Likelihood Ratio methods. Further genome-wide association study revealed seven associated SNPs that were related with intramuscular fat content and feed conversion ratio traits, respectively. Enrichment analysis suggested that the artificial selection regions harbored genes, such as *MSTN*, *SOD2*, *MC5R* and *CD83*, which are responsible for economic traits including lean muscle mass, fertility and immunization. Overall, this study found a series of candidate genes putatively associated with the breeding improvement of Duroc pigs and the polygenic basis of adaptive evolution, which can provide important references and fundamental information for future breeding programs.

## Introduction

Duroc, an older breed of domestic pig, was developed in America and formed the basis for many mixed-breed commercial boars after a long period of artificial selection. It is predominantly used as the terminal sires in pig industry, and is well known for its superior performance in growth, feed conversion efficiency, carcass and meat quality traits (Briggs and Briggs 1969). The artificial selection affecting these economic traits has left detectable selection signatures within the genome of modern Duroc pigs (Edea *et al.* 2017). Although the identification of selection signatures has been studied for decades on the basis of microsatellite and SNP arrays, there is now unprecedented opportunity for progress on fine mapping with the advent of large genome sequencing data sets on pig variation (Abbott 2012; Ai *et al.* 2015; Sabeti *et al.* 2002).

In general, most of the economic traits in commercial animals are quantitative and controlled by many genes with small effects (Khatkar *et al.* 2004). This quantitative genetics view is supported by most of the recent gene mapping researches, including the identification of selection signatures and genome-wide association studies (GWAS) (Eusebi *et al.* 2017; Ma *et al.* 2018; Pritchard *et al.* 2010; Ros-Freixedes *et al.* 2014). Therefore, genomic selection has become the main method in genetic improvement in important economic traits (Hayes *et al.* 2009). However, for traits such as meat quality and feed efficiency that are difficult and expensive to measurement, genomic selection is still limited by the sample size of the reference population. So, based on gene mapping, genetic improvement through marker-assisted selection is still an important alternative to improve these traits. Simultaneously, the development of genome selection methods, combined with the information of GWASs, will help further improve the accuracy of genetic evaluation (Zhang *et al.* 2014).

To address the growing demand for high quality pork and minimize pig breeding costs, the understanding of genomic architecture underlying pork quality and feed efficiency is valued by animal scientists and breeders. Intramuscular fat (IMF) content is one of the most important factors affecting pork quality. Based on marker-assisted selection, there is increasing interest in mapping some of the causal genes of IMF for its genetic improvement (Ros-Freixedes *et al.* 2014). Feed conversion ratio (FCR) is an important indicator for feed efficiency and is usually included in the selection index for genetic improvement in pig breeding (Do *et al.* 2014). Although a series of QTL associated with interesting economic traits were reported based on traditional QTL mapping, it is still scarce to investigate the genetic basis of IMF and FCR using genome-wide association study in pigs (Do *et al.* 2014; Hu *et al.* 2016; Ros-Freixedes *et al.* 2014; Sato *et al.* 2016).

In this study, we used paired-end Illumina sequencing to resequence the genomes of four purebred Durocs and used the genotyping by sequencing approach to characterize the genomes of 282 individuals from the same population. To identify the signatures of artificial selection, sequences from 23 purebred Durocs, 21 Asian wild boars (AWB), and 25 European wild boars (EWB) were downloaded from the most advanced publically available database. Two different statisticses, the Composite of Likelihood Ratio (CLR) and the fixation index (F_ST_), were applied to detect selection signatures. In addition, we also carried out GWAS to identify the key genes related with IMF and FCR that were changed during the breeding improvement of Durocs. We found strong signatures of selection left in Durocs during breed formation exemplified by several striking selective sweeps overlapping with some major QTLs. The results would provide useful information for those who are interested in further understanding the genetic basis of important commercial traits, and facilitate future breeding of Durocs to improve these traits through genomic selection.

## Material and Methods

### Ethics statement

All research involving animals was conducted under protocols (No. 5 proclaim of the Standing Committee of Hubei People’s Congress) approved by the Standing Committee of Hubei People’s Congress and the ethics committee of Huazhong Agricultural University in P. R. China. In addition, all experiments were performed in accordance with approved relevant guidelines and regulations.

### Animals, genome sequencing, quality checking and filtering

In this study, genomic DNA was extracted from ear tissues of each of 286 purebred Duroc pigs using a standard phenol-chloroform method. Among them, four individuals were selected to sequence the whole genomes. Paired-end sequencing libraries with an insert size around 350-bp were constructed for each sample. 2×150-bp paired-end sequencing was performed on an Illumina HiSeq X-ten platform at the BGI-Huada Genomics Institute in Shenzhen. The rest of individuals were genotyped by sequencing (GBS) on an Illumina HiSeq 2000 platform in Buckler Lab for Maize Genetics and Diversity at Cornell University (Elshire *et al.* 2011). In addition, we also downloaded the whole genome sequence data of 69 individuals from the EMBL-EBI database (https://www.ebi.ac.uk/), including 23 Duroc pigs, 21 Asian wild boars and 25 European wild boars (Supplementary Table S1). Quality control of sequence data applied the following criteria: reads with (i) > 10 bp aligned to the adapter with up to 10% mismatches, (ii) up to 10% unidentified nucleotides (N), (iii) > 50% bases having a phred quality less than 5 were removed, and (iv) Duplicate reads generated by PCR amplification in the library construction process were also removed.

### Read alignment and SNP calling

Clean reads passing quality control filter were aligned to the Sus scrofa reference genome (Sscrofa11.1) using the Burrows-Wheeler Aligner (BWA) software (Li and Durbin 2009). The reference genome sequence was indexed and the command ‘mem –t 8’ was used to find the suffix array (SA) coordinates of good hits for each individual read. SAMtools was then used to convert the SA coordinates into the best alignments in BAM format (Li *et al.* 2009). After alignment, ‘CreateSequenceDictionary’, ‘SortSam’ and ‘MarkDuplicates’ of Picard were separately used to indexing, sorting and removing potential PCR duplications (Li *et al.* 2009). The BAM files were indexed by Samtools. The ‘HaplotypeCaller’, ‘SelectVariants’ and ‘VariantFiltration’ of GATK with default parameters and SAMtools ‘mpileup’ module with the parameters as ‘-q 1 -ugf’ were used to call SNPs. Finally, the GATK ‘VariantFiltration’ module was used to exclude SNP calling errors in according to the following criteria (Mckenna *et al.* 2010). For GBS individuals, joint genotyping was performed by the GATK ‘GenotypeGVCFs’ module.

High quality SNPs with (i) coverage depth ≥ 4, (ii) RMS mapping quality ≥ 20, (iii) the distance of adjacent SNPs ≥ 5 bp, (iv) the missing ratio of samples within each population < 50%, and (v) the minor allele frequency (MAF) ≥ 0.01 were kept for further analysis. The SNPs that were used for GWAS need further apply quality control of Hardy–Weinberg equilibrium (*P* < 10e–6). To decrease the influence from genotype imputation, only SNPs with the missing ratio of samples within each population < 20% were used to detect selection signatures. Beagle software was used to impute the missing genotypes and infer haplotypes with default settings (Browning and Browning 2016).

### SNP annotation

We functionally annotated Single nucleotide variants (SNVs) with the gene-based annotation modules of ANNOVAR (Wang *et al.* 2010). Using ENSEMBL genes, we investigated where the SNVs are located in the regions of gene components.

### Linkage disequilibrium

We calculated the correlation coefficient (*r*^2^) for every pair of SNPs to measure the LD level in Duroc pigs and two wild boar populations using PLINK (Purcell *et al.* 2007). To visualize the LD decay in this analysis, the *r*^2^ values for 1000-bp distance bins were averaged and the corresponding figure was drawn by R script.

### Identification of artificial selection signatures

Identification of selection signatures is an important field of population genetics. To detect the selection signals accurately and objectively, a larger sample size is able to better reflect genomic patterns shaped by selection in the particular population. Therefore, all 27 whole-genome sequencing individuals were taken together to detect positive selection signatures referring to the result of principal components analysis (Supplementary Fig. S1). To identify artificial selection signatures, AWB and EWB were defined as the reference population, respectively.

In this analysis, two methods were employed to search for the evidence of artificial selection in two steps. The first step is positive selection detection, which was performed using the composite likelihood ratio (CLR) (Nielsen *et al.* 2005). The CLR method calculates the likelihood ratio of selection signals by comparing the spatial distribution of allele frequencies in an observed window to the frequency spectrum of the whole genome. SweepFinder was employed to calculate the CLR with a grid size of 25 kb resulting in a total of 90,588 CLR scores across the genome. In the second step, we identified the underlying artificial selection signals from the above detected positive selection signals by calculating the F_ST_ statistic for pairwise sites between the observed populations and the reference population (Weir and Cockerham 1984). The unbiased F_ST_ estimate proposed by Weir and Cockerham was used to measure the population differentiation, with values ranging from 0 (no differentiation) to 1 (complete differentiation). To produce comparable CLR and F_ST_ test results, single site scores for F_ST_ were averaged in non-overlapping windows of 25 kb resulting in a total of 90,322 and 90,411 F_ST_ scores across the genome when AWB and EWB were defined as the reference population, respectively. The empirical P-values were generated by genome wide ranking of F_ST_ and CLR values (Qanbari *et al.* 2014; Simianer *et al.* 2014). Finally, the windows that the scores of the statistics fell into the 98^th^ percentile were considered significant in CLR and F_ST_ methods, respectively. Note that the artificial selection signatures in this study were defined in the genomic region in which both CLR statistical value and the F_ST_ statistical value were greater than the cut-off value at the genome level.

### Genome-wide Association studies

In this analysis, all 282 castrated Duroc boars were treated similarly. All individuals were in good health and had the same body weight at the beginning of the experiment. The feed conversion ratio was calculated during the growth period from 30Kg to 100Kg, and at the end of the measurement, the intramuscular fat of each pig was determined by B ultrasound (Supplementary Table S2).

After quality control assessment, autosomes of 282 individuals genotyped by sequencing were used in two association studies for IMF and FCR traits, respectively. GEMMA was used to fit the model **y*=u+Ss+Xb+Wα+e***, where **y** is the phenotypes vector, ***u*** is the intercept, ***S*** is a design matrix of the fixed effects, ***s*** is the batches effect, ***b*** is the SNP effect, ***X*** is a design matrix for allele dosages for the imputed SNPs, ***W*** is an incidence matrix linking α to y, ***α*** is the additive genetic effect ∼ N (0, G*б*^2^*_a_*), where *б*^2^*_a_* is the additive genetic variance and G is the realized genomic relationship matrix that was estimated using genotype information, and **e** is the random residual term. To avoid double fitting of SNP effects efficiently, the test SNP and the other SNPs in the same chromosome were removed in constructing the G matrix each time based on the standardized relatedness matrix in GEMMA (Zhou and Stephens 2012).

Since Bonferroni correction is overly conservative especially when genetic data has high linkage disequilibrium, it may cause false negative results (Duggal *et al.* 2008). Therefore, a less conservative significance threshold of 1.03 × 10^−5^ (0.05/4,853) based on the SimpleM method was used to account for multiple tests in this analysis. A total of 4,853 independent tests were identified here that was in turn inferred by the number of principal components accounting for a 99% of the variance of the SNP matrix (Gao *et al.* 2008).

### Enrichment analysis for artificial selection signatures

Enrichment analysis was carried out for exploring the potential biological functions of genes located in putative artificial selection regions. It is involved all the selected genes in the 200 kb window around the significant signatures, which was determined by the linkage disequilibrium decay (Supplementary Fig. S2). Genes located in putatively selected regions were identified using the BioMart program (http://www.biomart.org/, Kasprzyk 2011), and then an enrichment analysis, which included the terms cellular component (CC), molecular function (MF), biological process (BP), and pathway analysis was performed for the identified genes using DAVID 6.7 (http://david.abcc.ncifcrf.gov/) (Huang *et al.* 2009).

### Data availability

The Illumina sequence reads are available in the NCBI Sequence Read Archive under the accession SRP158574. Supplemental files, the GBS data and phenotype data can be downloaded from Figshare (https://figshare.com/s/e45cc6d717cd498d5013). Supplemental material available at Figshare: https://doi.org/10.25387/g3.7077125.

## Results and Discussion

### Genome resequencing and genetic variation

After quality control, a total of 127.98 Gb of sequence data were generated on the basis of our four Duroc pigs, with the average sequencing depth of approximately 13-fold and the average genome coverage of 99.03%. As shown in Table 1, we identified 9,245,511 SNPs with an average density of 3.76 SNPs/kb. Then, we compared these SNPs that we found with those from the SNPs database that was built using 23 downloaded Duroc pigs. About 88% of the variants (8,205,625 SNPs) in our SNP data set were found in the SNPs database, whereas more than 11% (1,039,886 SNPs) of the variants that we identified were absent from the SNPs database (Supplementary Fig. S3). These novel SNPs substantially expand the database of Duroc genetic variants. Therefore, combining all 27 Duroc individuals together not only increased the sample size, but also significantly increased the SNPs density of the genome. The previous study indicates that a high marker density has positive effects on the identification of selection signatures (Ma *et al.* 2015).

**Table 1:** Summary and annotation of SNPs in Duroc pigs

Category			WGS		GBS
Sample size		27	4	23	282
Average depth (X)		12.10	12.79	11.98	16.92^1^
Average genome coverage (%)		96.56	99.03	96.13	0.56
Average Mapping rate (%)		99.67	99.66	99.67	95.95
High-quality base (Gb)		817.35	127.98	689.37	45.49
Q20 (%)		97.36	95.23	97.73	98.13
Q30 (%)		91.18	89.67	91.44	91.91
**Number of total SNP**		14,827,549	9,245,511	13,787,663	651,425
Upstream		92,785	56,487	85,634	4,094
UTR5		28,436	18,070	26,101	1,917
Exonic	Stopgain	545	265	504	215
	Stoploss	117	65	114	27
	Synonymous	66,285	41,379	61,367	5,663
	Nonsynonymous	44,660	26,352	41,243	10,212
	Unknown	152	33	144	215
Splicing		614	397	565	254
Intronic		5,496,375	3,459,359	5,108,598	192
UTR3		133,194	82,605	124,775	281,638
UTR5/UTR3		702	454	679	8,489
Downstream		98,207	59,700	91,145	53
Upstream/downstream		2,328	1,455	2,164	4,852
Intergenic		8,863,149	5,498,890	8,244,630	144

1 the population sequencing depth.

Combined, a total of 14,827,549 SNPs with an average density of 6.03 SNPs/kb were detected using 27 sequencing Duroc individuals. Among them, 8,863,149 were located in intergenic regions, 5,496,375 were in intronic regions and 111,759 were in exonic regions. Similarly, 45.49 Gb of sequence data were generated by GBS, with the population sequencing depth of approximately 17-fold and about 0.56% of the bases in the reference genome being covered by at least one reads. A total of 651,425 SNPs were identified in the 282 pig genomes, including 144 intergenic SNPs, 192 intronic SNPs and 16,332 exonic SNPs (Table 1). The SNPs identified by GBS were not evenly distributed across the genome, with an average density of 0.27 SNPs/kb (Supplementary Fig. S4). Similar genome annotation information of EWB and AWB herein is illustrated in Supplementary Table S3.

### Genome-wide artificial selection signatures

**Figure 1:**
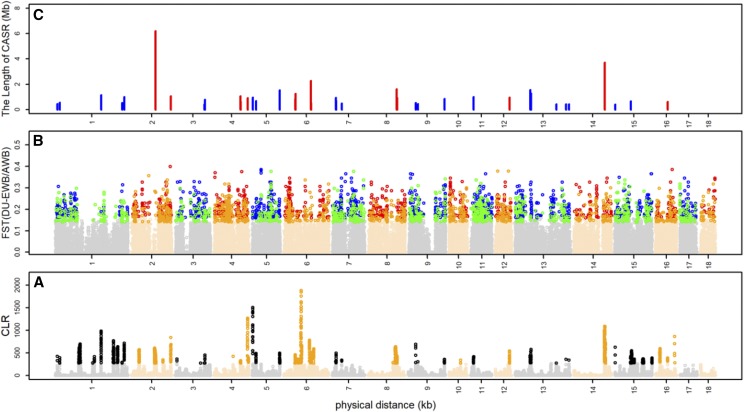
Summary of the genomic regions underlying artificial selection in Duroc pigs. (A) Manhattan plot based on CLR tests. (B) Manhattan plot based on F_ST_ tests, the points in blue and red (black and orange) represent scores when EWB (AWB) was treated as reference population. (C) The lines illustrate the positions and lengths of candidate artificial selection regions (CASR).

To detect positive selection, the CLR scores were calculated using an identical grid size across the genome. We focused the analyses on windows for which the scores of the statistics fell in the top 2^nd^ percentile. As shown in Supplementary Fig. S5, the threshold value is almost greater than the largest CLR scores in the wild populations. In general, we expected a limited amount of artificial selection for commercial traits occurred in the wild populations. Out of 90,588 sliding windows, 1,811 windows were identified as the potential selection signatures in the Duroc population. Then, the windows within a 200kb fragment around the potential selection signatures are merged and the genomic regions were defined as candidate selection regions (CSRs). Correspondingly, a total of 70 fragments, spanning lengths of 81.40 Mb and covering 3.3% of the genome, were identified as CRSs in Duroc pigs (Figure 1A).

To further identify the artificial selection signatures, F_ST_ statistics were used to detect the differentiation between Duroc and wild pigs. We calculated F_ST_ per site and averaged them in non-overlapping 25 kb windows across the genome. The windows that the scores of the statistics fell in the top 2^nd^ percentile were considered as significant. Out of 90,322 sliding windows, 1,806 windows were identified as the potential selection signatures and fall into 758 CRSs when AWB was treated as reference population. Similarly, Out of 90,411 sliding windows, 1,804 windows were identified as the potential selection signatures and fall into 684 CRSs when EWB was treated as reference population (Figure 1B).

In this analysis, the overlapping candidate selection regions detected by CLR and F_ST_ were defined as candidate artificial selection regions (CASR). Collectively, a total of 38 CASRs, spanning lengths of 40.48 Mb and covering 1.62% of the genome, were identified in Duroc pigs through compared with the wild populations (Figure 1C, Supplementary Table S4). In general, the adaptive evolution of important traits, due to human-driven or natural select, would leave a number of selection signatures in genomic regions, where should harbor the corresponding causal genes. Therefore, the candidate selection regions should be major enriched in genic regions. To test this hypothesis, we identified a total of 246,868 SNPs in the 38 candidate artificial selection regions, of which 143,978 (57.918%) were intergenic, 97,287 (39.136%) were intronic and 1,924 (0.78%) were exonic. Comparing the distribution of SNPs between candidate artificial selection regions and the whole genome, it suggested that there was no predictable pattern in where those selection regions were located (Supplementary Table. S5). This seems to indicate that selection not only plays a role in the gene regions, but also in other regulatory elements of the genome.

### Candidate selection regions harboring loci associated with two economic traits

**Figure 2:**
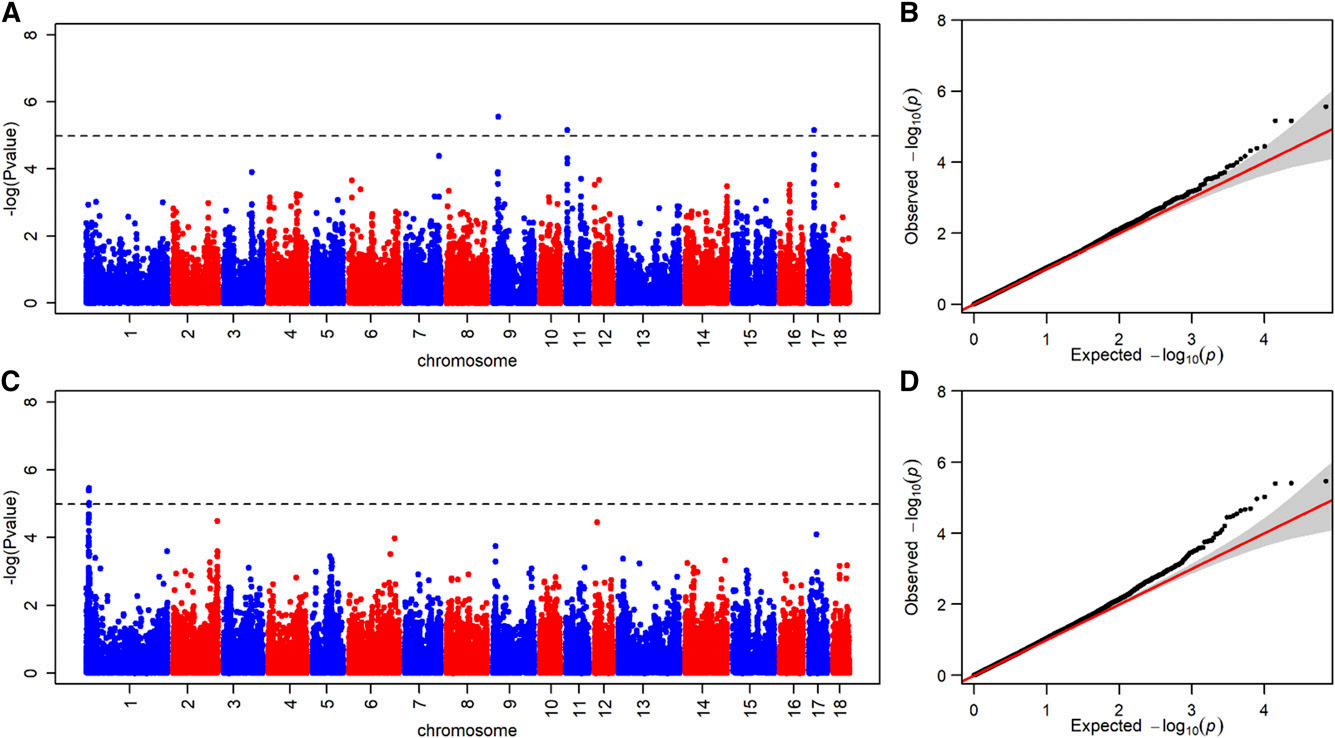
Visualization of the signals revealed by genome-wide association analyses for IMF and FCR traits. (A, C) Manhattan plots present the association of the imputed SNPs with the IMF and FCR traits in 282 Duroc individuals, respectively. (B, D) The corresponding quantile-quantile plots.

Genome-wide association studies have already been proven effective to reveal the underlying genetics of economic traits (Meyer *et al.* 2016; Visscher *et al.* 2017). If the underlying genetic basis of those domestication traits has been improved by recent artificial selection, the genomic regions that harbored the QTNs revealed through GWAS should overlap with the candidate selection regions. In this analysis, we performed mixed-model association analysis using GBS data of 35,303 high-quality markers. Although the small sample size limited our power, we identified seven associated loci with a less conservative significance threshold (−log10(p-value) ≥4.98), including four SNPs for IMF trait and three SNP for FCR trait (Figure 2). Comparing seven associated loci with the candidate selection region, we found that a series of selection signatures were located around the associated loci for both traits (Supplementary Table S6).

Because of concerns that the sample size for GWAS is too small to make meaningful scientific conclusion, we investigated the results of GWAS for IMF and FCR traits using a random sampling of 250 individuals, and the process is repeated 10 times. Among them, the similar results of GWAS for IMF and FCR traits have been replicated with smaller sample size, respectively (Supplementary Fig. S6, S7). In addition, we further check genotype-class frequencies and phenotypic means for seven significant SNPs. As shown in Supplementary Table S7, we can clearly observe the trend of phenotypic changes with three genotypes. The results suggested that the SNPs passing the less conservative significance threshold are still promising in this study.

For FCR trait, an associated SNP was close to the 16.375-16.425 Mb selection region on SSC9 and the QTL of ‘time spent feeding’ was reported to be overlapping with this region. For IMF trait, 4 associated SNPs were found close to the 7.1-9.0 Mb selection regions on SSC1. After scanning the pig QTLdb (https://www.animalgenome.org), we found that these four SNPs were also overlapping with ‘the drip loss’ and ‘the stearic acid content’, respectively (Hu *et al.* 2016).

**Figure 3:**
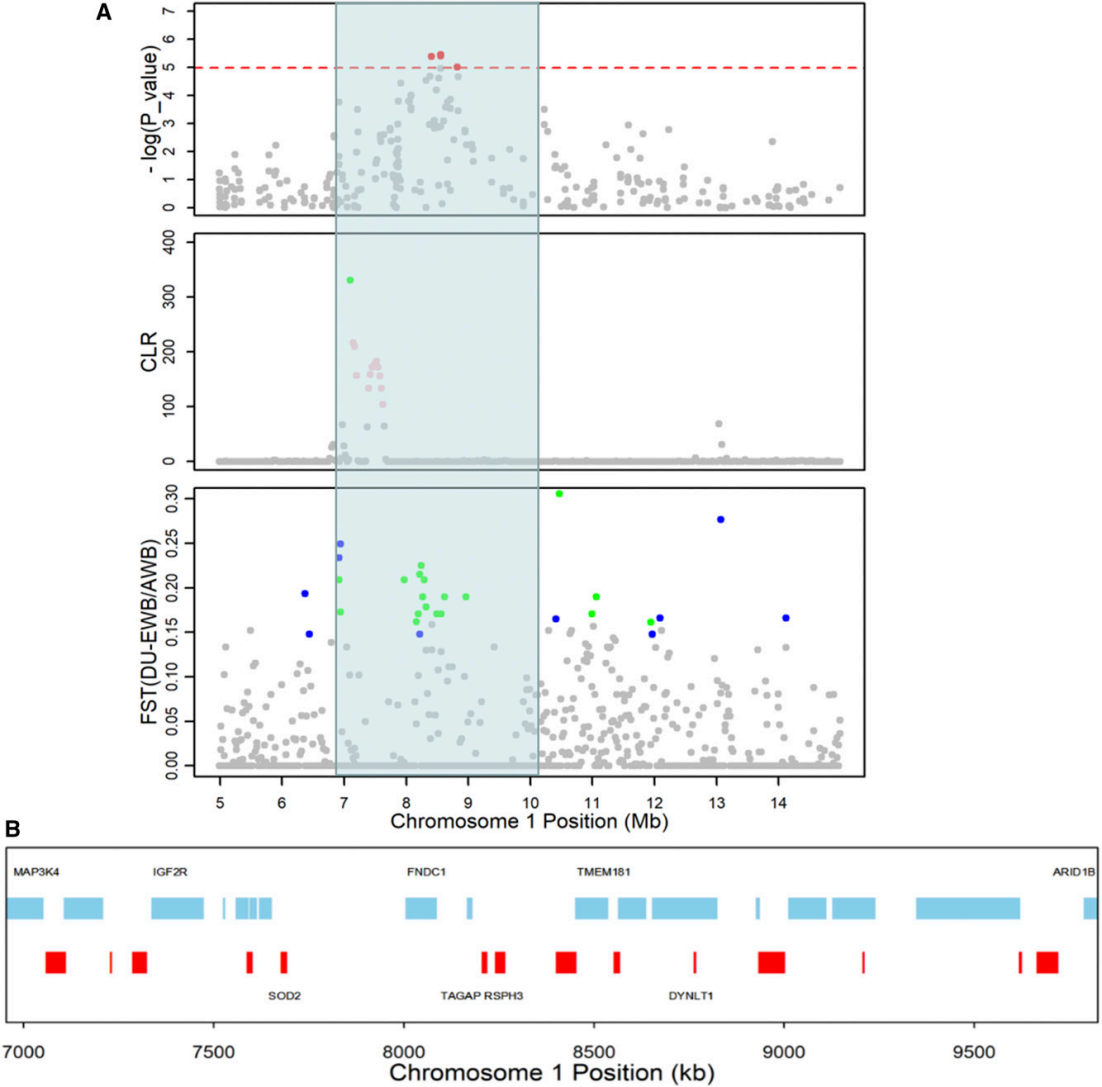
Comparison of GWAS and candidate artificial selection regions (CASR) on SSC1 in Duroc. (A) The top Manhattan plot shows GWAS results for IMF trait. The middle plot shows the distribution of CLR scores calculated in sliding windows. The bottom plot is the distribution of F_ST_ scores when AWB (blue) and EWB (green) were treated as reference population, respectively. The red line is the significance threshold. (B) The positions of 31 gene models in this region are indicated: skyblue, minus-strand genes; red, positive-strand genes. The detailed gene list can be found in Supplementary Table S8.

We highlighted the 7.00-10.00 Mb regions around the most significant SNP on SSC1 (Figure 3), where a series of CLR and F_ST_ scores exceeded the significance threshold and several harboring genes, such as *IGF2R*, *TMEM181*, *SOD2* and *TAGAP*, were responsible for sex determination, growth, muscle and bone development (Supplementary Table. S8). Among them, one possible positional candidate gene, insulin like growth factor 2 receptor (*IGF2R*), serves *IGF2* turnover in IGF signal mediated process (Ludwig *et al.* 1996) and *IGF2* is known as a major gene that influences the meat quality of pigs (Clark *et al.* 2014).

### Go terms, pathways and candidate genes for artificial selection

To further investigate the genetic basis of improved economic traits, a total of 371 genes overlapping with all 38 candidate artificial selection regions in Duroc pigs were found and the corresponding orthologous genes from human were used to perform an enrichment analysis by DAVID 6.7 (https://david.ncifcrf.gov/) (Huang *et al.* 2009). The results indicated that genes related with a number of terms previously implicated in breed improvement were present within or close to these sweep regions.

Among them, a set of genes overlapping artificial selection regions were enriched for the ability of immunity, which mainly included ‘GO:0042267∼natural killer cell mediated cytotoxicity’ (6 genes), ‘GO:0019882∼antigen processing and presentation’ (6 genes) and ‘hsa04650:Natural killer cell mediated cytotoxicity’ (6 genes) (Supplementary Table S9). Additionally, we also found one significant candidate artificial selection regions that overlapped with *CD83* gene, which contributes to T lymphocyte proliferation (Pinho *et al.* 2014) (Table 2). Due to the high density and selection intensity of rearing animals in the breeding farm, the artificial selection effect is undoubted to play an indispensable role on selecting innate immunity genes indirectly. Six genes with strong selection signatures are significantly enriched in ‘GO:0043651∼linoleic acid metabolic process’ and the *LPIN2* gene that plays a significant role in fat deposition was found to fall into the 102-104.275 Mb artificial selection regions on SSC6 in Duroc, suggesting that these genes may have been selected during the breeding of lean pigs (Table S2, S9).

**Table 2:** Some candidate genes overlap with the potential regions of artificial selection in Duroc pigs

Chr.	Pos. (Mb)^1^	P-value. (method)^2^	Gene	Gene function
1	7679352..7691724	0.01;0.001(AWB); 0.007(EWB)	*SOD2*	Fertility (Kwiatkowska *et al.* 2017)
2	51102757..51128346	0.048;0.008(EWB)	*WNT9A*	Chondrogenesis (Später *et al.* 2006)
2	88122788..88255282	0.005;<0.001(AWB)	*HOMER1*	Muscle development (Hao *et al.* 2017)
2	88021572..88098522	0.007;<0.001(AWB)	*JMY*	Development of porcine embryos (Lin *et al.* 2015)
2	88391280..88507344	0.004;<0.001(AWB)	*CMYA5*	Carcass trait and meat quality (Xu *et al.* 2011)
6	96322276..96323795	0.003	*MC5R*	Back fat thickness, lipid metabolism, exocrine function, proinflammatory activity (Switonski *et al.* 2013)
6	103997590..104055816	0.009;0.007(AWB)	*NDC80*	Fertility (Wei *et al.* 2014)
6	103578544..103728152	0.008;0.007(AWB)	*LPIN2*	Back-fat thickness (He *et al.* 2009)
7	10359211..10387002	0.017;0.005(AWB); 0.006(EWB)	*CD83*	Enhances T lymphocyte proliferation (Pinho *et al.* 2014)
7	75161831..75168634	0.027;0.001(AWB); 0.004(EWB)	*FITM1*	Fat-deposition-related traits (li *et al.* 2010)
7	75241058..75255701	0.027;0.001(AWB); 0.004(EWB)	*DHRS4*	Meat quality (Hwang *et al.* 2017)
9	35187561..35200085	0.032;<0.001(AWB);0.006(EWB)	*CASP1*	Fertility (Ashworth *et al.* 2010)
12	50454063..50491164	0.036;0.007(AWB)	*SPNS2*	Hearing (Chen *et al.* 2014)
15	94623526..94628440	0.046, 0.009(EWB)	*MSTN*	Lean muscle mass (Baati *et al.* 2017)

1 This column presents the position of candidate genes which overlap with or close to the potential regions of artificial selection.

2 This column presents the genome-wide P-values of sweep statistics.

In addition to those significant Go and Pathway terms, a set of artificial selection signatures with extreme *P*-value coincide with a cluster of genes involved in meat quality, growth, fertility and so on (Table 2). An interesting selection signature was located on SSC15 (104.325-104.8 Mb) and close to the *MSTN* gene, which was associated with lean muscle mass and played an important role in the process of muscle development (Baati *et al.* 2017). As a commercial pig breed of economic importance, leanness has consistently been considered as an objective trait of Durocs breeding. Correspondingly, growth rates and meat quality would also be considered and designed in the breeding program. In this study, *WNT9A* gene associated with chondrogenesis and *CMYA5* gene associated with carcass length and meat quality were overlapping with the strong selection signatures. *MC5R* gene, an established sweep in previous studies, was found close to the 97.625-98.725 Mb regions on SSC6, which was not only associated with back fat thickness but also played an important role in proinflammatory activity (Switonski *et al.* 2013). The *HOMER1* gene that is overlapped with the 86.55-92.75 Mb artificial selection regions on SSC2 is associated with muscle differentiation and calcium homeostasis (Hao *et al.* 2017). Note that a series of genes, including *SOD2*, *JMY*, *NDC80* and *CASP1*, overlapping with artificial selection regions in this analysis were associated with the reproductive traits. In comparing with its wild ancestors, litter size, fetal weight and the other fertility traits would change for adapting human-driven selection. These results indicated that the improvement of fertility and other complex traits may due to the polygenic basis, rather than be caused by only a few critical loci.

## Conclusions

In summary, this study detected signatures of artificial selection and identified a number of loci associated with some important economic traits, like IMF and FCR, which provided an important resource in future Durocs breeding programs. SNP annotation implied that there was no predictable distribution in where those artificial selection regions were located in genome. Enrichment analyses suggested that the polygenic basis may be a reasonable explanation for the phenomenon, that many different genes associated with the economic traits have often been selected during the improvement and breed formation. The application of combining sweep analysis and genome-wide association analysis are effective in mapping commercial important genes, especially to the data with small sample size caused by the expensive measurement traits.
